# Initial experience with laparoscopic radical antegrade modular pancreatosplenectomy for left-sided pancreatic cancer in a single institution: technical aspects and oncological outcomes

**DOI:** 10.1186/s12893-016-0200-z

**Published:** 2017-01-07

**Authors:** Eun Young Kim, Tae Ho Hong

**Affiliations:** Department of Hepato-biliary and Pancreas Surgery, Seoul St. Mary’s Hospital, College of Medicine, The Catholic University of Korea, 222, Banpo-daero, Seocho-gu, Seoul, 06591 Republic of Korea

**Keywords:** Distal pancreatectomy, Laparoscopy, Left-sided pancreas cancer

## Abstract

**Background:**

Laparoscopic surgery has been performed less frequently in the era of pancreatic cancer due to technical difficulties and concerns about oncological safety. Radical antegrade modular pancreatosplenectomy (RAMPS) is expected to be helpful to obtain a negative margin during radical lymph node dissection. We hypothesized that it would also be favorable as a laparoscopic application due to unique features.

**Methods:**

Fifteen laparoscopic RAMPS for well-selected patients with left-sided pancreatic cancer were performed from July 2011 to April 2016. Five trocars were usually used, and the operative procedures and range of dissection were similar to or the same as those of open RAMPS described by *Strasberg*. All medical records and follow-up data were reviewed and analyzed.

**Results:**

All patients had pancreatic ductal adenocarcinoma. Mean operative time was 219.3 ± 53.8 min, and estimated blood loss was 250 ± 70 ml. The length of postoperative hospital stay was 6.1 ± 1.2 days, and postoperative morbidities developed in two patients (13.3%) with urinary retention. The median number of retrieved lymph nodes was 18.1 ± 6.2 and all had negative margins. Median follow-up time was 46.0 months, and the 3-year disease free survival and overall survival rates were 56.3% and 74.1%, respectively.

**Conclusion:**

Our early experience with laparoscopic RAMPS achieved feasible perioperative results accompanied by acceptable survival outcomes. Laparoscopic RAMPS could be a safe and oncologically feasible procedure in well-selected patients with left-sided pancreatic cancer.

## Background

While the oncologic feasibility of laparoscopic surgery has been accepted for colon, stomach, and liver malignancies [1–5], only a few surgeons have performed laparoscopic surgery in the era of pancreatic cancer due to its fastidiousness for adequate dissection and the safety margin [6]. However, several studies have reported that a laparoscopic approach for pancreatic malignancies can result in favorable outcomes [1–5, 7, 8], and the need for discussion has emerged.

In 2003, Strasberg described an approach to resect left-sided pancreatic cancer called radical antegrade modular pancreatosplenectomy (RAMPS), which is a novel procedure that includes a horizontal dissection plane from right-to-left and radical resection of regional lymph nodes based on anatomic drainage of the pancreas. RAMPS has been performed more frequently with the expectation that it could be helpful to obtain negative tangential margins and a favorable survival rate [9–11]. RAMPS has some unique features that are favorable for application to laparoscopic surgery. The direction of dissection (from right-to-left) in RAMPS is familiar to operators with conventional laparoscopic distal pancreatectomy experience for benign or borderline malignant tumors, and this RAMPS feature helps the operator feel more comfortable during laparoscopic RAMPS.

The aim of this study was to describe the technical aspects of our laparoscopic RAMPS experience and present survival outcomes of laparoscopic RAMPS in selected patients with left-sided pancreatic cancer.

## Methods

Fifteen laparoscopic RAMPS for well-selected patients with left-sided pancreatic cancer were performed from July 2011 to April 2016 at the Department of Surgery, Seoul St. Mary’s Hospital. All patients were evaluated preoperatively using abdominal computed tomography (CT) scans and magnetic resonance cholangiopancreatography to accurately identify the location of the cancer. A positron emission tomography scan was used to detect the distant metastasis. Laparoscopic RAMPS was selectively applied to cases diagnosed as left-sided pancreatic cancer that was less than stage T3 without distant metastasis or peritoneal seeding on the preoperative imaging study. Cases in which we were unable to secure a safety margin from a major vessel, such as the superior mesenteric artery (SMA) or vein or celiac axis, were excluded from the laparoscopic approach. Cases exceeding the T4 stage or in which adjacent organs, such as the stomach, colon, or kidney, had been invaded, except the left adrenal gland, were also treated using an open method. The study was approved by the ethics committee of the Seoul St. Mary’s Hospital (IRB No. KC15RISI0939). Patients provided written informed consent from each participant, and the procedures were in compliance with Helsinki Declaration.

### Operative technique

**Fig. 1:**
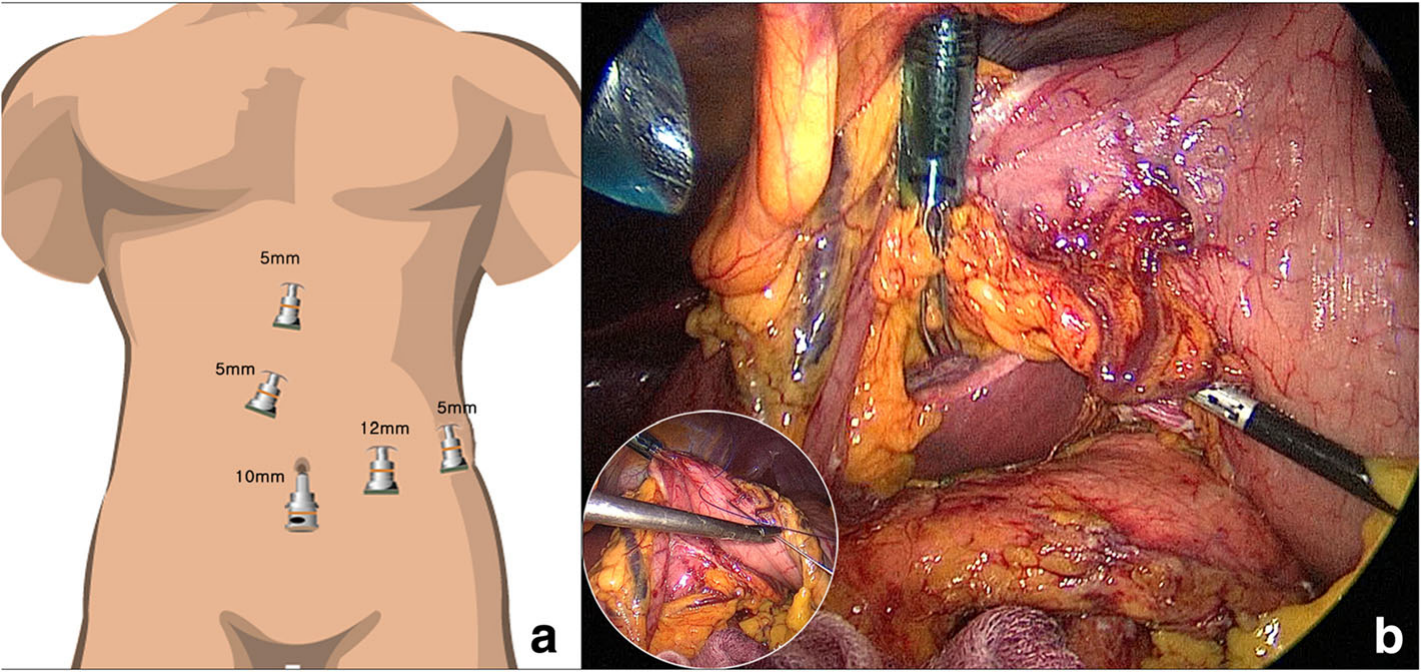
Trocar positions (**a**) and intraoperative view of the working space for laparoscopic radical antegrade modular pancreatosplenectomy (RAMPS) (**b**)

**Fig. 2:**
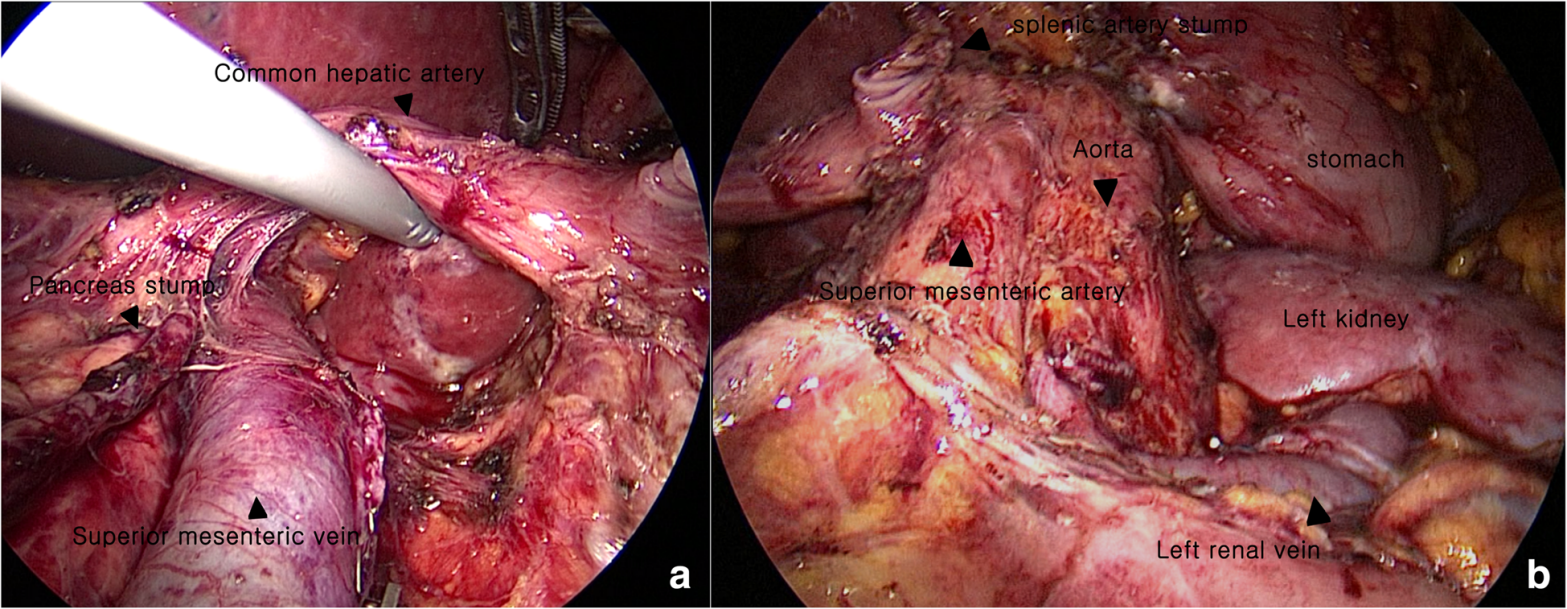
Completion of lymph node dissection. Lymph nodes along the common hepatic artery (CHA) and gastroduodenal artery (GDA) were removed after sufficient mobilization of the pancreas by dissecting the tissue around the upper border of the pancreas (**a**). The lymph nodes dissected around the celiac axis, the superior mesenteric artery, the left adrenal gland, and Gerota’s fascia were completely resected in a case of posterior radical antegrade modular pancreatosplenectomy (RAMPS) (**b**)

The patient was placed with legs apart in the supine position and tilted to the right side in the reverse Trendelenburg position. The operator was positioned to the right side of the patient, and the first assistant and scrub nurse stood on the opposite side. The second assistant held the laparoscope and was positioned between the patient’s legs. We created a pneumoperitoneum through the umbilicus using an open technique and a 10-mm trocar under the direct vision. Intra-abdominal pressure was maintained at about 12 mmHg with carbon dioxide. Five trocars were usually used (Fig. 1a); one 10-mm umbilicus trocar for the laparoscope, one 12-mm trocar on the left midclavicular line for the left hand of the operator, and three 5-mm trocars (one on the mid-epigastrium for the right hand of the first assistant, one at the subxiphoid for stomach traction, and the other at the left flank for the right hand of the operator). Operative procedures and range of dissection were similar to or the same as those of the open method described by Strasberg et al. [9]. We inspected the intraperitoneal cavity carefully after entry. The lesser sac was entered after dividing the gastro-colic and gastro-splenic ligaments close to the stomach to remove the gastrosplenic nodes. We generally hung the stomach using direct sutures to the abdominal wall to create a working space under the stomach (Fig. 1b). The lymph nodes along the common hepatic artery (CHA) and gastroduodenal artery were removed after sufficient mobilization of the pancreas through dissecting the tissue around the upper border of the pancreas (Fig. 2a). The right gastric artery was divided routinely for proper dissection of the lymph node along the CHA and gastroduodenal artery during open RAMPS, but we did not need to divide the right gastric artery routinely during the laparoscopic approach because the laparoscope could be passed in the space created beneath the stomach. The pancreatic neck was elevated off the superior mesenteric vein (SMV) and portal vein (PV) and a window was created between the posterior surface of the pancreas and the confluence of the SMV, PV and splenic vein. The pancreatic neck was transected with straight endoscopic linear staples (Echelon Endopath™ Stapler, Ethicon Endo-Surgery, Inc., Cincinnati, OH, USA) after sufficient peri-firing compression. Staple size depended on the hardness or thickness of the pancreas. Lymph nodes around the celiac axis were dissected to expose the origin of the splenic artery (Fig. 2b). The splenic artery was ligated and divided using a laparoscopic ligation system (Hem-o-lok® Ligation System, Teleflex Medical, Boston, MA, USA) and the splenic vein was also resected using the endo-GIA (white cartilage). The lymph nodes were dissected medial-to-lateral and the resection range was up to the diaphragmatic crus, down to the left renal vein, and to the left lateral portion of the aorta on the posterior side. The dissection continued more laterally to the left of Gerota’s fascia, and the inferior mesenteric vein was divided after detaching the distal pancreas with the underlying fascial layer from the retroperitoneum. The operator used either the anterior or posterior RAMPS procedure to maximize the chance of achieving a negative tangential margin. The decision was based on the principles emphasized by Strasberg et al. [9]; therefore, the left adrenal gland and Gerota’s fascia were completely resected concomitantly during posterior RAMPS (Fig. 2b). After completely resecting the distal pancreas with *en bloc* lymph node dissection, the specimen was bagged and retrieved through the umbilical port site with minimal extension. Two closed suction drains were used; one for the pancreatic stump through the 5-mm port mid-epigastric incision and the other for the splenectomy site through the left flank port site.

### Postoperative management and outcome assessment

The medical records and follow-up data of all patients were reviewed retrospectively for patient demographics, operative results (operative time, estimated blood loss, and type of RAMPS), tumor characteristics (tumor differentiation, tumor size, number of retrieved lymph nodes, TNM stage, and margin status), postoperative outcomes (pain score, length of postoperative hospital stay, start of soft diet, postoperative complications, and mortality), and follow-up data. Tumor size and differentiation and the number of retrieved lymph nodes were recorded from the pathology report. Margin status included negative (RO) and positive margin resection (R1 or R2), and these margins included the superior and inferior borders and the posterior surface of the specimen. The transected surface at the neck of pancreas and all tangential margins of the specimen that were not covered with peritoneum were marked with ink on the back table after retrieving the specimen to assess margin status. TNM cancer stage was evaluated based on the AJCC Caner Staging Manual, 7^th^ edition. We checked postoperative pain using a visual analog scale (VAS) from 0 (no pain) to 10 (worst pain imaginable) on postoperative days 1, 3, 5, and 7, and length of stay was estimated from the day of the operation to the day of discharge. Postoperative complications were reviewed and analyzed according to the Clavien–Dindo classification [12]. A postoperative pancreatic fistula was graded as A, B, or C based on the International Study Group of Pancreatic Fistula definition [13]. We also assessed the incidence of digestive complications, such as prolonged diarrhea defined as loose stools for at least 4 weeks after surgery. Wound infection was established as any complication of a trocar site with tenderness or erythema requiring opening, drainage, or antibiotics although a seroma or hematoma was not considered a wound infection. Postoperative mortality was defined as mortality within 30 days of surgery or within the same hospital stay as the surgery. Patients with acceptable postoperative physical status were treated with adjuvant chemotherapy by the oncologist under our institutional policy.

## Results

### Patient demographics and perioperative outcomes

The demographics and perioperative outcomes of laparoscopic RAMPS are summarized in Table 1. Six men (40%) and nine women (60%) were included, with mean age of 68.1 ± 9.2 years (range, 50–79) and mean body mass index of 21.9 ± 3.8 kg/m^2^ (range, 16.4–28.1 kg/m^2^). Mean operative time was 219.3 ± 53.8 min (range, 119–305 min) and mean estimated blood loss was 250 ± 70 mL (range, 150–400 mL). Three patients (20%) received intraoperative transfusions, no case was converted to open surgery, and no postoperative mortality occurred. Posterior RAMPS was performed in eight cases (53.3%) because preoperative CT scans showed that the tumor had penetrated the posterior capsule of the pancreas. The VAS pain score decreased gradually over time from 4.1 ± 1.8 on postoperative day 1 to 2.5 ± 1.1, 1.5 ± 1.1, and 0.7 ± 0.6 on postoperative days 3, 5, and 7, respectively. It took a mean of 2.6 ± 0.6 postoperative days (range, 2–4 days) for patients to return to an oral diet, and the mean number of postoperative hospital days was 6.1 ± 1.2 (range, 5–9 days). Postoperative urinary retention complications developed in two patients (13.3%) who were treated conservatively. No case of digestive complications, such as prolonged diarrhea or ileus, or pulmonary complications, such as atelectasis or pleural effusion, occurred that required additional management.

**Table 1:** Results of all patients who underwent laparoscopic radial antegrade modular pancreaticosplenectomy for left-sided pancreatic cancer

Characteristics	Total (*n* = 15)
(a) Patient demographics and perioperative outcomes	
Age (range, yr)	68.1 ± 9.2 (50–79)
Sex (M/F)	7/8
BMI (range, kg/m^2^)	21.9 ± 3.8 (16.4–28.1)
ASA class (%)	
Class I	5 (33.3)
Class II	7 (46.7)
Class III	3 (20)
Operative procedure (%)	
Anterior RAMPS	7 (46.7)
Posterior RAMPS	8 (53.3)
Conversion to laparotomy (%)	0
Operative time (range, min)	219.3 ± 53.8 (119–305)
Estimated blood loss (range, ml)	250 ± 70 (150–400)
Intraoperative transfusion (%)	3 (20)
Postoperative pain^a)^	
POD 1	4.1 ± 1.8
POD 3	2.5 ± 1.1
POD 5	1.5 ± 1.1
POD 7	0.7 ± 0.6
Postoperative hospital stay (range, day)	6.1 ± 1.2 (5–9)
Return to oral diet (range, day)	2.6 ± 0.6 (2–4)
Overall complications (%)	2 (13.3)
urinary retention	2 (13.3)
Hospital mortality (%)	0
(b) Oncologic outcomes	
Tumor differentiation (%)	
well differentiated	3 (20)
moderately differentiated	11 (73.3)
poorly differentiated	1 (6.7)
T stage (%)	
T2	2 (13.3)
T3	13 (86.7)
N stage (%)	
N0	9 (60)
N1	6 (40)
TNM staging (%)	
stage IB	1 (6.7)
stage IIA	8 (53.3)
stage IIB	6 (40)
Tumor size (range, cm)	3.8 ± 1.8 (1.8–4.5)
Count of retrieving lymph node (range)	18.1 ± 6.2 (10–30)
R0 resection (%)	15 (100)
Negative tangential margin (%)	15 (100)
Recurrence (%)	4 (26.7)
Metastasis (%)	3 (20)

^a)^ estimated by visual analog scale (VAS) score

### Oncological outcomes

All 15 patients were diagnosed with ductal adenocarcinoma on the pathology report. Mean tumor size was 3.8 ± 1.8 cm (range, 1.8–4.5 cm), and mean length of the resected pancreas was 10.1 ± 1.8 cm (range, 6.7–13.2 cm). Tumor differentiation stages were three well differentiated (20%), eleven moderately differentiated (73.3%), and one poorly differentiated (6.7%). The median number of lymph nodes retrieved was 18.1 ± 6.2 (range, 10–30), and six patients (40%) had malignant-positive lymph nodes. Thirteen patients (86.7%) had T3 tumors that had invaded the peripancreatic tissue from outside the pancreatic capsule. All of these cases achieved a negative tangential margin and R0 resection on the permanent pathological report.

### Survival and follow-up outcomes

**Fig. 3:**
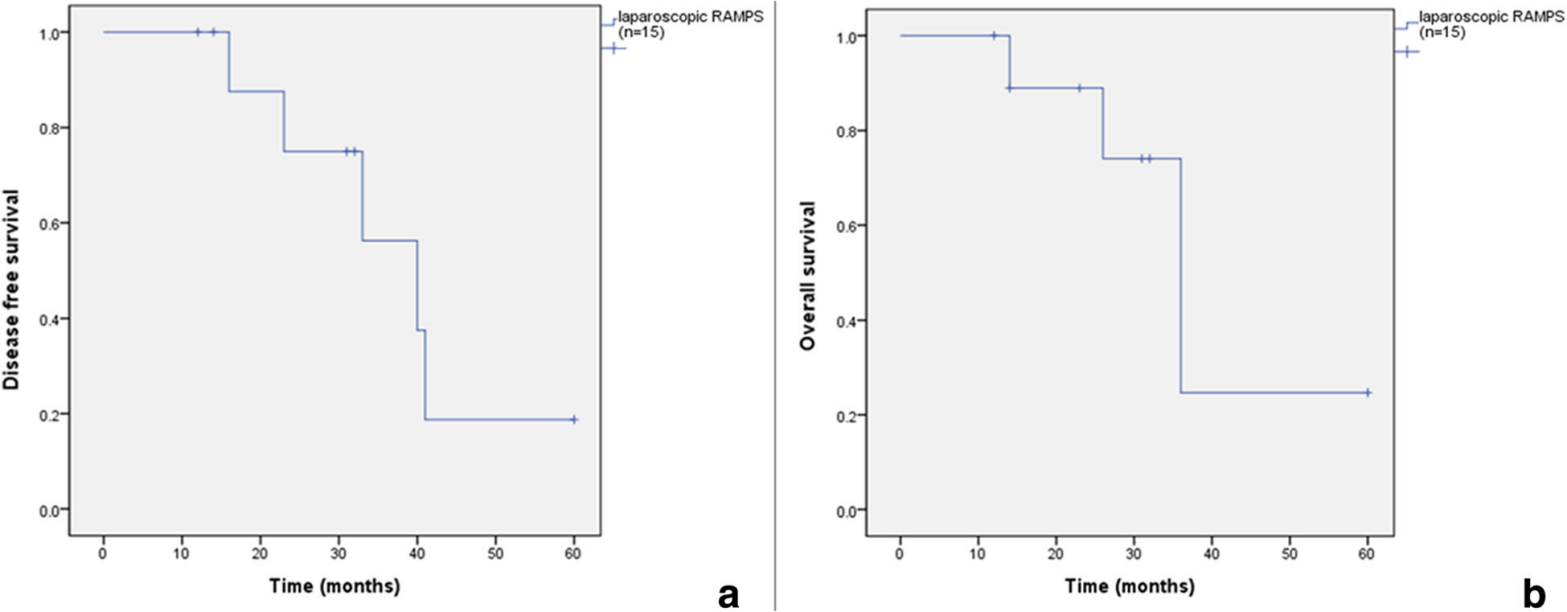
Kaplan–Meier survival curve of laparoscopic radical antegrade modular pancreatosplenectomy (RAMPS) in well-selected cases of left-sided pancreatic cancer (*n* = 15); (**a**) disease free survival, and (**b**) overall survival rates

Mean and median follow-up times were 46.6 and 46.0 months, respectively. Four patients (26.7%) developed disease recurrence; two patients (13.3%) had a local recurrence in the pancreatic bed, one patient (6.7%) had a recurrence around the celiac axis and the other patient (6.7%) had a recurrence around the SMA. One patient with a pancreatic bed recurrence presented with carcinomatosis peritonei 25 months after surgery. The 1-year disease free survival rate (DFS) was 100%, and the 3-year DFS was 56.3%. Median survival was 40.0 months, and 1-year and 3-year overall survival (OS) rates were 100% and 74.1%, respectively (Table 2). Five patients (33.3%) died 16–41 months after surgery. The Kaplan–Meier survival curve of laparoscopic RAMPS is presented in Fig. 3.

**Table 2:** Survival outcomes of laparoscopic radical antegrade modular pancreatosplenectomy (RAMPS) in well-selected cases of left pancreatic cancer (*n* = 15)

	n	Disease free survival (%)			Overall survival (%)		
		1-year	2-year	3-year	1-year	2-year	3-year
Lap. RAMPS	15	100	75.0	56.3	100	88.9	74.1

## Discussion

Laparoscopic surgery has been widely accepted due to its minimally invasive approach and advantages, such as less bleeding, smaller transfusion requirement, and shorter incisions with less pain. Consequently, laparoscopic surgery has been expanded to various fields of surgery and these advantages also applied to laparoscopic RAMPS. Conventional open RAMPS usually requires a long midline abdominal incision or left subcostal incision that could cause severe pain and easily develop infection. Prolonged wound pain can be associated with difficulties coughing or expectoration and disturb ambulation and can cause postoperative morbidities, such as atelectasis and ileus, which prevent early recovery of normal activities. In our study, postoperative pain improved rapidly over time, and no postoperative morbidities, such as wound infection, atelectasis, or ileus, were observed. We also showed favorable outcomes in terms of starting an oral diet and length of hospital stay [1, 7, 14–17]. We expect that the early return to an oral diet improved nutritional status, which would shorten recovery and advance the commencement of adjuvant treatment.

Although the safety and oncological feasibility of laparoscopic surgery for pancreatic cancer remains controversial, several studies have reported favorable outcomes of laparoscopic approaches to the pancreatic cancer as experience has accumulated [3, 18, 19]. We have applied laparoscopic RAMPS to selected cases of left-sided pancreatic cancer since 2011 and our early experience showed acceptable oncological outcomes with adequate lymph node harvest and negative margin status. Our results are mainly attributed to the properties of RAMPS and feasibility for a laparoscopic approach. First, RAMPS does not require complex reconstruction of an anastomosis unlike pancreaticoduodenectomy, so the entire procedure is relatively simple, which is favorable for a laparoscopic approach. Second, the dissection proceeds from right to left, which is familiar to operators with conventional laparoscopic distal pancreatectomy experience for benign or borderline malignancies. An operator accustomed to laparoscopic distal pancreatectomy can easily adapt to a RAMPS laparoscopic approach. The familiar manipulations during the surgery are helpful for reducing burden on the operator, which and produces fewer tissue injuries. Third, RAMPS uses a more objective dissection plane, and some difficulties determining the overall anatomic structure at a glance can occur during laparoscopic surgery because the magnified view is provided for a limited area, unlike open surgery. This disadvantage could be complemented during RAMPS because the objective dissection plane allows for an easier operative process without the need to capture the whole anatomy. We believe these RAMPS characteristics contributed to the safe and feasible outcomes in laparoscopic approach.

The right-to-left dissection with a magnified view during laparoscopic RAMPS provides a posterior dissection plane that can assist acquiring a sufficient margin. Actually, our results revealed successful tumor margin status and survival outcomes over the long-term. We obtained a negative tangential margin and R0 resection in all cases. We also achieved favorable 1- and 3-year DFS (100% and 56.3%, respectively) and 1- and 3-year OS rates (100% and 74.1%, respectively). Additionally, trocar site metastasis and wound recurrence which are concerns for malignancy during laparoscopic surgery, were not observed in our study. The oncological outcomes of our study and those of previous reports using RAMPS are summarized in Table 3 [10, 19–24].

**Table 3:** Previously reported laparoscopic radical antegrade modular pancreatosplenectomy (RAMPS) or open RAMPS trials or series

Publication	No. of patients	Year of publication	Mean tumor size (cm)	Operative time (min)	EBL (ml)	Length of hospital stay (day)	Count of retrieved lymph nodes	Margin status, RO (tangential) (%)	Median survival (month)
Open RAMPS									
Strasberg et al. [10]	23	2007	5.1	378	630	11	15	87 (91)	21
Mitchem et al. [19]	47	2011	4.4	244	744	11.3	18	81 (89)	26
Chang et al. [20]	24	2012	4.1	305	-^a^	-^a^	21	92 (92)	18.2
Park et al. [21]	38	2014	3.1	210	325	11.5	14	89.4 (-)^a^	24.6
Kitagawa et al. [22]	24	2014	3.5	387	371	11.5	28	88 (92)	-^b^
Laparoscopic RAMPS									
Choi et al. [23]	4	2012	-^a^	390	475	7	9	100 (100)	24
Lee et al. [24]	12	2014	2.8	324	446	12	11	100 (100)	60
Kim et al. ^c^	10	2015	4.1	290	284	9	20	100 (100)	40

^a^ Data not described in this report

^b^ Five-year overall survival rate was 53%

^c^ Current study

The mean number of retrieved lymph nodes in this study was 18.1 ± 6.2, which is comparable with previous RAMPS reports (Table 3). The magnified angular laparoscopic view facilitated the delicate manipulation around lymph nodes (Fig. 2). Moreover, we suspended the posterior wall of the stomach using direct sutures to the abdominal wall, and we also used a 30°-sloped laparoscope. These allowed sufficient working space beneath the stomach. As a result, we were able to approach the lymph nodes around the gastroduodenal artery and common hepatic artery more easily without routinely resecting the right gastric artery, unlike during open RAMPS.

Our surgical outcomes should be interpreted with caution because of limitations. We are only presenting our early experience of laparoscopic RAMPS and did not compare the results with those of conventional open RAMPS. However, the surgical outcomes for left-sided pancreatic cancer are shown in oncologic safety and survival outcomes results, accompanied by reduced pain and a shorter hospital stay. A comparative study composed of more samples and with conventional open RAMPS should be performed to confirm the feasibility of laparoscopic RAMPS for left-sided pancreatic cancer.

## Conclusions

We achieved feasible survival outcomes accompanied by successful negative resection margins and radical lymph node dissection during laparoscopic RAMPS. Our data show reduced postoperative pain, which hastened recovery. We suggest that laparoscopic RAMPS is a safe and oncologically feasible procedure in well-selected cases of left-sided pancreatic cancer. However, a further prospective comparative trial with open RAMPS should be conducted.

## Abbreviations

CHA: Common hepatic artery
CT: Computed tomography
DFS: Disease free survival
OS: Overall survival
PV: Portal vein
RAMPS: Radical antegrade modular pancreatosplenectomy
SMA: Superior mesenteric artery
SMV: Superior mesenteric vein.
